# Measuring personal beliefs and perceived norms about intimate partner violence: Population-based survey experiment in rural Uganda

**DOI:** 10.1371/journal.pmed.1002303

**Published:** 2017-05-23

**Authors:** Alexander C. Tsai, Bernard Kakuhikire, Jessica M. Perkins, Dagmar Vořechovská, Amy Q. McDonough, Elizabeth L. Ogburn, Jordan M. Downey, David R. Bangsberg

**Affiliations:** 1 Chester M. Pierce, MD Division of Global Psychiatry, Massachusetts General Hospital, Boston, Massachusetts, United States of America; 2 Harvard Medical School, Boston, Massachusetts, United States of America; 3 Mbarara University of Science and Technology, Mbarara, Uganda; 4 MGH Global Health, Massachusetts General Hospital, Boston, Massachusetts, United States of America; 5 Bloomberg School of Public Health, Johns Hopkins University, Baltimore, Maryland, United States of America; 6 Last Mile Health, Boston, Massachusetts, United States of America; 7 Oregon Health Sciences University–Portland State University School of Public Health, Portland, Oregon, United States of America; Medical Research Council, SOUTH AFRICA

## Abstract

**Background:**

Demographic and Health Surveys (DHS) conducted throughout sub-Saharan Africa indicate there is widespread acceptance of intimate partner violence, contributing to an adverse health risk environment for women. While qualitative studies suggest important limitations in the accuracy of the DHS methods used to elicit attitudes toward intimate partner violence, to date there has been little experimental evidence from sub-Saharan Africa that can be brought to bear on this issue.

**Methods and findings:**

We embedded a randomized survey experiment in a population-based survey of 1,334 adult men and women living in Nyakabare Parish, Mbarara, Uganda. The primary outcomes were participants’ personal beliefs about the acceptability of intimate partner violence and perceived norms about intimate partner violence in the community. To elicit participants’ personal beliefs and perceived norms, we asked about the acceptability of intimate partner violence in five different vignettes. Study participants were randomly assigned to one of three survey instruments, each of which contained varying levels of detail about the extent to which the wife depicted in the vignette intentionally or unintentionally violated gendered standards of behavior. For the questions about personal beliefs, the mean (standard deviation) number of items where intimate partner violence was endorsed as acceptable was 1.26 (1.58) among participants assigned to the DHS-style survey variant (which contained little contextual detail about the wife’s intentions), 2.74 (1.81) among participants assigned to the survey variant depicting the wife as intentionally violating gendered standards of behavior, and 0.77 (1.19) among participants assigned to the survey variant depicting the wife as unintentionally violating these standards. In a partial proportional odds regression model adjusting for sex and village of residence, with participants assigned to the DHS-style survey variant as the referent group, participants assigned the survey variant that depicted the wife as intentionally violating gendered standards of behavior were more likely to condone intimate partner violence in a greater number of vignettes (adjusted odds ratios [AORs] ranged from 3.87 to 5.74, with all *p <* 0.001), while participants assigned the survey variant that depicted the wife as unintentionally violating these standards were less likely to condone intimate partner violence (AORs ranged from 0.29 to 0.70, with *p*-values ranging from <0.001 to 0.07). The analysis of perceived norms displayed similar patterns, but the effects were slightly smaller in magnitude: participants assigned to the “intentional” survey variant were more likely to perceive intimate partner violence as normative (AORs ranged from 2.05 to 3.51, with all *p <* 0.001), while participants assigned to the “unintentional” survey variant were less likely to perceive intimate partner violence as normative (AORs ranged from 0.49 to 0.65, with *p*-values ranging from <0.001 to 0.14). The primary limitations of this study are that our assessments of personal beliefs and perceived norms could have been measured with error and that our findings may not generalize beyond rural Uganda.

**Conclusions:**

Contextual information about the circumstances under which women in hypothetical vignettes were perceived to violate gendered standards of behavior had a significant influence on the extent to which study participants endorsed the acceptability of intimate partner violence. Researchers aiming to assess personal beliefs or perceived norms about intimate partner violence should attempt to eliminate, as much as possible, ambiguities in vignettes and questions administered to study participants.

**Trial registration:**

ClinicalTrials.gov NCT02202824.

## Abstract 2

In this population-based experiment in rural Uganda, Alexander Tsai and colleagues identify survey questions that more accurately measure the attitudes of men and women to intimate partner violence.

## Author summary

**Why was this study done?:**

- Personal beliefs and perceived norms about the acceptability of intimate partner violence are important determinants of the health risk environment for women.
- For nearly two decades, the Demographic and Health Surveys have served as an important source of information on beliefs about the acceptability of intimate partner violence. The survey questions present study participants with hypothetical scenarios that might be encountered by a husband and wife (or cohabiting couple) and then ask study participants to evaluate whether they believe the husband would be justified in behaving violently toward the wife.
- Important studies have suggested limitations in the accuracy with which these types of questions measure beliefs about the acceptability of intimate partner violence.

**What did the researchers do and find?:**

- We embedded a randomized survey experiment in a population-based household survey conducted in Mbarara, Uganda, a rural region located in the southwestern part of the country.
- Study participants were randomly assigned to one of three survey conditions that manipulated contextual details about the wife’s intentions underlying her behavior that purportedly prompted the violent behavior from the husband.
- We found that contextual details about the intentionality of the wife’s violations of gendered standards of behavior shifted participants’ personal beliefs and perceived norms about the acceptability of intimate partner violence.

**What do these findings mean?:**

- Researchers aiming to assess personal beliefs or perceived norms about intimate partner violence should attempt to eliminate, as much as possible, ambiguities in vignettes and questions administered to study participants.
- Follow-up studies aim to elucidate whether and how the questions on beliefs about intimate partner violence in the Demographic and Health Surveys should be changed.

## Introduction

In national studies conducted throughout sub-Saharan Africa, survey data indicate that there is widespread acceptance of intimate partner violence by both men and women [1–4]. Men who personally believe in traditional concepts of masculinity and in the acceptability of intimate partner violence are more likely to be perpetrators of sexual violence [5]. Considered in the aggregate, in the context of a population, individual beliefs about intimate partner violence collectively define norms about intimate partner violence. In the classic typology introduced by Cialdini et al. [6], descriptive norms describe “what is typical or normal.” The social context of these gender-unequal norms—the personal beliefs held by most individuals within a population or some other salient social grouping—also has important implications for population health and mental health [7]. Women are more likely to experience intimate partner violence if they live in areas characterized by gender-unequal norms about intimate partner violence [8,9] and when they and their partners report concordant beliefs about the acceptability of intimate partner violence [4]. Exposure to violence has well-established adverse impacts on health and mental health [10–15,80]. Therefore, accurate measurement of personal beliefs about intimate partner violence has important implications for understanding the health risk environment for women.

The health risk environment is shaped not only by actual exposures to intimate partner violence but also by perceptions about the acceptability of intimate partner violence. Being in a social environment where gender-unequal norms are prominent may compromise women’s reproductive health and decision-making [7,16,17,81], irrespective of any direct exposure to violence [18,19]. That this phenomenon has been replicated in multiple contexts suggests that perceived norms about intimate partner violence—i.e., individual perceptions about what *others in the community believe* about the acceptability of intimate partner violence—are also relevant for understanding health behaviors and health risk. Consistent with this hypothesis, an observational study in the US found that men who perceived that intimate partner violence was normative were themselves more likely to perpetrate violence against their partners [20]. Similar findings have been documented with regard to excessive alcohol consumption [21,22], HIV transmission risk behavior [23,24], and HIV testing [25]: these studies all documented that study participants who perceived these behaviors or attitudes as being more normative were also more likely to engage in these behaviors or hold such attitudes themselves.

For nearly two decades, the Demographic and Health Surveys (DHS)—nationally representative surveys conducted worldwide—have served as an important source of information on beliefs about the acceptability of intimate partner violence. Typically these beliefs are elicited by providing study participants with a hypothetical vignette and then asking whether they believe violence against the woman portrayed in the vignette is justified under the circumstances [26]. Importantly, a cross-country analysis of DHS data showed that minor deviations in survey wording may account for substantial cross-country variation in the extent to which intimate partner violence is deemed acceptable [27]. Furthermore, two qualitative studies of Bangladeshi women suggested that affirmative responses to DHS-style questions may reflect their perceptions of prevailing norms or their own assumptions about causal attributions (in terms of assigning fault to perpetrators versus victims of violence), rather than reflecting their personal beliefs about the acceptability of intimate partner violence [28,29].

These multiple lines of inquiry suggest important limitations in the accuracy with which the DHS measure attitudes toward intimate partner violence. One large random-digit dial survey conducted in the US state of California found that manipulations of different contextual variables heavily influenced survey respondents’ causal attributions [30], but there has been no experimental evidence from sub-Saharan Africa that can be brought to bear on this issue. To address this important gap in the literature, we embedded a randomized survey experiment in a population-based household survey conducted in rural Uganda. Uganda generally provides legal protections for women and has undertaken to comply with international and regional human rights laws [31]. However, violence against women is common throughout the country [32]. The objective of our study was to determine the extent to which contextual information about the intentionality of the wife’s violations of gendered standards of behavior affects participants’ responses to questions about their personal beliefs and perceived norms about intimate partner violence.

## Methods

### Study setting and population

The study site was Nyakabare Parish in Uganda’s Mbarara District, located approximately 260 km southwest of the capital city, Kampala. The local economy is largely based on subsistence agriculture, and food and water insecurity are common [33,34]. Although the parish is only 20 km from the Mbarara town center, for most residents the cost of transportation is a difficult economic barrier to overcome and serves to reinforce their geographic isolation [35,36]. Through an iterative process involving field investigations and conversations with local officials, Nyakabare was chosen as the study site because it was smaller than many other parishes in the region, thereby facilitating our ability to capture a whole-population sample; the village leaders welcomed the idea of a population-based household survey; and the village leaders described relatively little involvement in service delivery or other development activities by non-governmental organizations.

Approximately 3 mo prior to survey administration, we conducted a population census within the parish by approaching individuals living in the parish and by obtaining information about individuals from neighbors and other social contacts. In total, we enumerated 1,551 eligible persons living in 758 households: eligible persons were adults aged 18 y and older (or emancipated minors aged 16–18 y) who considered Nyakabare their primary place of residence and who were capable of providing consent. We excluded minors younger than 18 y of age, with the exception of emancipated minors; persons who did not consider Nyakabare their primary place of residence, e.g., persons who happened to be visiting Nyakabare at the time of the survey or who owned a home in Nyakabare but spent most of their time outside the parish; persons with whom research staff could not communicate, e.g., due to deafness, mutism, or aphasia; and persons with psychosis, neurological damage, acute intoxication, or other cognitive impairment (all of which were determined informally in the field by non-clinical research staff in consultation with a supervisor).

### Experimental procedures

The Computer Assisted Survey Information Collection (CASIC) Builder software program (West Portal Software Corporation, San Francisco, California) served as the basis for the survey, which was administered in the field with the use of laptop computers. We first wrote survey questions in English, translated them into Runyankore, and then had them back-translated into English to verify the fidelity of the translated text. The translation and back-translation was an iterative process involving in-depth consultation and pilot testing with 18 key informants.

The primary outcomes were participants’ personal beliefs about the acceptability of intimate partner violence and perceived norms about intimate partner violence in their communities. Survey questions presented study participants with hypothetical scenarios that might be encountered by a typical Ugandan married or cohabitating couple in this region. The five scenarios involved a wife going out without her husband’s permission, neglecting to keep the children well fed, arguing with her husband in public, refusing to have sexual intercourse with her husband, and not preparing her husband’s meal on time (see Box 1; survey questions were administered sequentially in the order shown in Text A of S1 Appendix). To elicit participants’ *personal beliefs* about intimate partner violence, they were asked whether or not they thought violent behavior by a husband directed toward his wife was justified in any of these five different scenarios. Response options for these questions were as follows: “agree,” “disagree,” “refuse to answer,” and “don’t know.” To elicit *perceived norms* about intimate partner violence, study participants were administered the same vignettes except they were instead asked about the extent to which they thought other people in the village would think violent behavior was justified. These questions were also informed by previously published studies of perceived norms about heavy alcohol use and other health risk behaviors [37,38]. We specified “your village” so that all participants would have a similar fixed, unambiguous reference group [39]. Response options for the five questions about perceived norms followed a four-point Likert-type scale: “all or almost all, for example, at least 90% of people in your village,” “more than half but fewer than 90% of people in your village,” “fewer than half but more than 10% of people in your village,” and “very few or none, for example, less than 10% of people in your village.” We initially used briefer response options that did not reference specific percentages, but the percentage amounts were added after extensive piloting suggested their addition would improve study participants’ comprehension of the questions.

**Box 1. Examples of survey questions to elicit personal beliefs and perceived norms about the acceptability of intimate partner violence**

**Survey question**

**Personal belief**

In your personal opinion, do you agree or disagree with the following statement?

**Perceived norm**

How many other people aged 18 years or older who stay in your village, not including yourself, do you think would agree with the following statement?

**Example scenario: Wife going out without her husband’s permission**

**DHS-style survey variant (version 1)**

Little contextual detail provided: “A husband is justified in hitting or beating his wife if she goes out without telling him or asking his permission.”

**“Intentional” survey variant (version 2)**

Wife depicted as intentionally violating gendered standards of behavior: “Imagine a situation in which a wife goes out to visit some friends or relatives, just for fun, without asking her husband’s permission or telling him where she is going. In this situation, the husband would be justified in hitting or beating his wife.”

**“Unintentional” survey variant (version 3)**

Wife depicted as unintentionally violating gendered standards of behavior: “Imagine a situation in which a wife is home alone, and the husband is away at work. Someone comes to tell her that her mother is very ill, so she goes to her parents’ home without asking her husband’s permission and stays the night. In this situation, the husband would be justified in hitting or beating his wife upon her return.”

**Fig 1:**
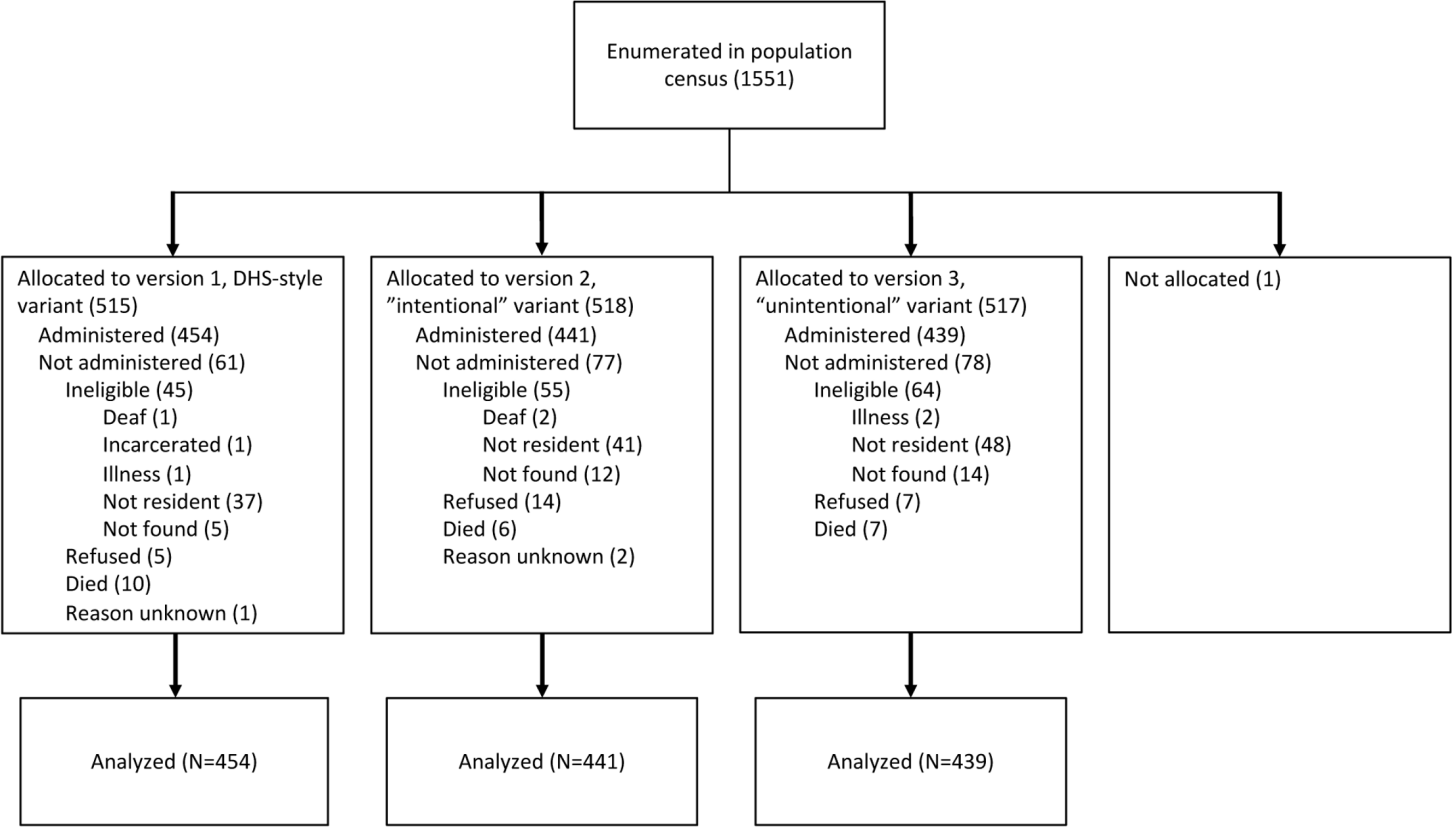
Consolidated Standards of Reporting Trials (CONSORT) flow diagram depicting enrollment, allocation to survey condition, follow-up, and data analysis. DHS, Demographic and Health Surveys.

Eligible study participants were randomly assigned to one of three survey conditions in a parallel group design as described in Fig 1. Each of the three survey conditions entailed the use of one of three different instruments for eliciting five personal beliefs and five perceived norms about the acceptability of intimate partner violence. The questions in the DHS-style survey variant (version 1) provided little contextual detail about the circumstances that purportedly prompted the violent behavior by the husband. These questions most closely approximate those administered in the DHS. The other two versions of the instrument manipulated specific contextual details about the intentionality of the wife’s behavior. The questions in the “intentional” survey variant (version 2) depicted the wife as intentionally violating gendered standards of behavior that might be expected of her. The questions in the “unintentional” survey variant (version 3) provided details that depicted the wife as unintentionally violating these standards of behavior.

Assignment to the three survey conditions in a 1:1:1 allocation ratio was determined centrally according to a computer-generated random schedule. Neither the research assistants administering the questionnaires nor the study participants were aware of the survey conditions to which the study participants had been assigned. (It should be noted, however, that the research assistants were not blinded. Thus, even though research assistants were unaware a survey experiment was being conducted, they may have perceived differences in the survey questions being administered to different study participants.) To avert chance imbalances by sex and geographical location [40], we generated 16 separate randomization schedules for subsets of participants defined by strata of sex and village of residence. The experimental procedures for this study were registered with ClinicalTrials.gov (NCT02202824).

### Sample size calculation

The sample size for this study was bounded by the population census. All eligible participants in the population were eligible for inclusion. Assumptions for the power calculation were derived from the 2011 Uganda Demographic and Health Survey: among women surveyed, the mean number of vignettes (out of five) for which violent behavior by a husband directed toward his wife was thought to be justified was 1.48 (variance, 4.00). A sample size of approximately 515 participants per survey condition would yield 84% power to detect a 25% relative difference in the mean number of vignettes for which intimate partner violence was thought to be justified (e.g., 1.48 versus 1.85) [41,42].

### Hypotheses

Cognitive interviews and qualitative data from Bangladesh [28,29] suggest that study participants may more frequently endorse the acceptability of intimate partner violence when survey vignettes depict women as intentionally violating gendered standards of behavior. These qualitative findings are consistent with social psychologists’ attribution theory models, which endeavor to explain how observers assign intentionality and responsibility, either implicitly or explicitly, and how these assignments influence their behaviors and moral evaluations [43,44]. A somewhat related literature in political science has shown how issue framing—variations in emphasis or salience that are placed on different aspects of a particular issue, including causal attributions—can manipulate public opinion [45–49]. For example, Iyengar [50] showed that study participants who viewed news stories emphasizing the structural drivers of poverty were much less likely to enumerate dispositional explanations for homelessness.

Based on these findings, we hypothesized that the extent to which the contextual details depict women as intentionally violating gendered standards of behavior would be associated with the extent of participant agreement with the acceptability of intimate partner violence. Specifically, we hypothesized that the survey variant depicting the wife as intentionally violating gendered standards of behavior would yield the greatest proportion of agreement, while the survey variant depicting the wife as unintentionally violating these standards would yield the lowest proportion of agreement. We further hypothesized that the DHS-style variant (which lacks contextual details about intentional versus unintentional violations of gendered standards of behavior) would be associated with an intermediate amount of agreement.

Additionally, we hypothesized that the effects would differ by sex. In a qualitative study from Bangladesh, Schuler et al. [51] employed cognitive interviews to better understand the mental processes underlying men’s and women’s responses to questions eliciting their personal beliefs about the acceptability of intimate partner violence. They found that women were much more likely to change their responses during the course of a cognitive interview, reflecting greater sensitivity to contextual nuances in the questions. Therefore, we hypothesized that the differences between the survey variants would be greater for women than for men.

### Statistical analysis

Covariate balance was assessed in two ways. First, among the participants who were successfully interviewed, we employed chi-squared tests to assess for balance on individual covariates (age, sex, educational attainment, marital/cohabiting status, and village) across the survey conditions. Second, we conducted an omnibus test for joint orthogonality [52] by fitting a multinomial logistic regression model to the data with survey condition as the dependent variable and the five covariates listed above as explanatory variables. We then used a Wald-type F-test to test the joint hypothesis that the regression coefficients were equal to zero.

The analysis was prespecified prior to data collection. To model the primary outcomes (number of vignettes [out of five] in which the study participant personally believed intimate partner violence to be justified and the number of vignettes in which the participant perceived intimate partner violence as being normative), our initial intent was to use negative binomial regression. Based on reviewer comments received after submission, we selected a multivariable proportional odds regression model [53,54]. The total number of vignettes was specified as the dependent variable and a three-level categorical variable for survey condition was specified as the primary explanatory variable of interest. For the five questions about personal beliefs, the omnibus outcome variable was simply the total sum of “agree” responses (with “disagree” responses grouped together with missing and indeterminate responses and all coded as 0). For the five questions about perceived norms, the omnibus outcome variable was the total sum of items for which participants responded “all or almost all” or “more than half.” To ensure accurate confidence intervals that accounted for the stratified randomization scheme, we adjusted the treatment estimates for sex and village by including them as covariates in the regression model. For both outcomes, the omnibus Wald test by Brant [55] suggested that the data differed significantly from the primary model assumption of proportional odds. In the analysis of personal beliefs, the proportional odds assumption was rejected (χ^2^ = 73.1, df = 40, *p =* 0.001); the omnibus result was driven by heterogeneous odds ratios for the survey effect across cutoff points. In the analysis of perceived norms, the proportional odds assumption was not rejected on the omnibus test (χ^2^ = 43.6, df = 40, *p =* 0.32), but inspection of the individual odds ratios across cutoff points revealed heterogeneous odds ratios for assignment to the “intentional” survey variant only (χ^2^ = 10.7, df = 4, *p =* 0.03).

Given these findings, we therefore fitted multivariable partial proportional odds regression models for the primary analysis [56]. The partial proportional odds regression model differs from the proportional odds regression model by permitting selected regression coefficients to vary across the logit equations. In the models applied in our analysis, we constrained all of the regression coefficients to be equal across the logit equations except for those corresponding to the survey conditions, which were permitted to vary (given that they were responsible for the violation of the proportional odds assumption). We employed robust estimates of variance corrected for clustering at the village level [57–59]. To assess the extent to which effects differed by sex of the participant, we fitted the same regression models with a sex-by-survey-condition product term (after confirming that the interaction terms did not violate the proportional odds assumption).

To interrogate the robustness of our findings, we conducted three post hoc sensitivity analyses. First, we examined each of the items separately to determine the extent to which the survey effects were consistent across items. In all of these analyses, the dependent variable was dichotomous, but we employed a Poisson model with cluster-correlated robust estimates of variance [57–59] so that the exponentiated regression coefficients could be interpreted as relative risks [60,61]. Second, because interviewer–respondent sex concordance may affect disclosure of sensitive material [62–64], we refitted the primary regression models including the sex of the interviewer and a product term for sex of the interviewer and sex of the participant (i.e., sex concordance) as covariates (after confirming that these additional covariates did not violate the proportional odds assumption). Third, because the omnibus outcome variables assume equal weights for the component variables, we constructed alternative outcome variables using principal components analysis to determine the weights [65]. This method is a technique that can be applied to a set of variables to extract the orthogonal linear combinations of the variables that represent the common information (in this case, a latent construct measuring belief in gender-unequal norms). Intuitively, the first principal component is simply the linear combination of all the variables that captures the largest amount of information that is common to the variables. The weights are not grounded theoretically but rather empirically. We extracted the first principal components and labeled them as our dependent variables of interest (“intimate partner violence beliefs index” and “intimate partner violence perceived norms index”), which were then used in linear regression models.

### Ethical and safety considerations

Each eligible person was approached in the field, typically at their home or (less frequently) their place of employment, by a research assistant who spoke the local language (Runyankore) and who requested their participation in the study. The survey was framed in general terms as a study about the social lives and health of residents of Nyakabare Parish, not as a study about attitudes toward intimate partner violence. For persons who expressed potential interest, the study was described in detail, and their written informed consent to participate was obtained. If there were literacy reasons why a written signature was not appropriate, study participants were permitted to indicate consent with a thumbprint. All research assistants received in-depth training on how to administer surveys for gathering sensitive information, including instructions on how to temporarily halt the survey if another person came within earshot. The research assistants also received two additional training courses, one from The AIDS Support Organization on how to handle study participant reports of intimate partner violence, receive the information sensitively and professionally, and provide appropriate referrals, and one from a Ugandan counseling psychologist on managing sensitive disclosures by study participants.

We solicited feedback on the study design from a community advisory board comprised of eight community leaders, including four women and the district community development officer. Ethical approval for all study procedures was obtained from the Partners Human Research Committee of Massachusetts General Hospital and the Institutional Review Committee of Mbarara University of Science and Technology. Consistent with national guidelines, we received clearance for the study from the Uganda National Council for Science and Technology and from the Research Secretariat in the Office of the President.

## Results

### Study population and characteristics

Of the 1,551 adults initially identified in the population census and randomly assigned to one of the three survey variants, we successfully interviewed 1,334 of them from June 3, 2014, to August 12, 2015 (Fig 1). Comparing the 1,334 successfully interviewed study participants with the 217 persons who were randomized but not interviewed, there were no statistically significant differences by sex, village of residence, or altitude of residence (*p*-values ranged from 0.09 to 0.15); we otherwise had a very limited ability to analyze differences given that no other data were collected on persons who were not successfully interviewed. Of the 515 participants assigned to the DHS-style survey variant, 454 were successfully interviewed (88%); of the remainder, 45 were subsequently deemed to be ineligible, ten died prior to survey administration, five refused, and one was not surveyed for unknown reasons. Of the 518 participants assigned to the intentional survey variant, 441 were successfully interviewed (85%); of the remainder, 55 were subsequently deemed to be ineligible, 14 refused, six died, and two were not surveyed for unknown reasons. Of the 517 participants assigned to the unintentional survey variant, 439 were successfully interviewed (85%); of the remainder, 64 were subsequently deemed to be ineligible, seven refused, and seven died. One participant was enumerated in the census but moved away shortly thereafter and was not allocated to any of the survey variants.

Summary characteristics for the sample, stratified by survey condition, are displayed in Table 1. The mean age was 41.5 y (standard deviation, 16.9 y), and women comprised a slight majority of study participants (756 [57%]). Most participants were married or cohabiting with a domestic partner (885 [66%]). Chi-squared tests revealed no statistically significance differences in covariates across the survey conditions (*p*-values ranged from 0.58 to 1.00). The omnibus test for joint orthogonality did not reject the null hypothesis (χ^2^ = 3.98, *p =* 1.00), suggesting that the randomization procedures did indeed achieve covariate balance across the survey conditions.

**Table 1:** Characteristics of the sample (*n =* 1,334).

Characteristic	Survey variant		
	DHS style	Intentional	Unintentional
**Women, *n* (percent)**	256 (56%)	254 (58%)	246 (56%)
**Age, mean (standard deviation)**	41.8 (17.1)	41.5 (16.5)	41.2 (17.2)
**Educational attainment, *n* (percent)**			
None	83 (18%)	89 (20%)	73 (17%)
Some primary (P1–P6)	140 (31%)	136 (31%)	148 (34%)
Completed primary (P7–P8)	110 (24%)	108 (24%)	98 (22%)
More than primary (S1–S6 or more)	121 (27%)	108 (24%)	120 (27%)
**Married or cohabiting, *n* (percent)**	299 (66%)	285 (65%)	301 (69%)

### Experimental findings

The omnibus variables for personal beliefs and perceived norms were correlated with each other but were not so closely correlated that they presented redundant information (Spearman’s ρ = 0.50). Across survey conditions, there was a negligible proportion of refusals and indeterminate (“don’t know”) responses to the questions about personal beliefs (less than 2%, depending on the outcome variable). The unadjusted means and proportions implied large effect sizes (Table 2). For the questions about personal beliefs, the mean (standard deviation) number of items where intimate partner violence was endorsed as acceptable was 1.26 (1.58) among participants assigned to the DHS-style survey variant, 2.74 (1.81) among participants assigned to the survey variant depicting the wife as intentionally violating gendered standards of behavior, and 0.77 (1.19) among participants assigned to the survey variant depicting the wife as unintentionally violating these standards. Responses to the questions about perceived norms followed a similar pattern (Table 3).

**Table 2:** Personal beliefs about the acceptability of intimate partner violence, unadjusted estimates by survey condition (*n =* 1,334).

Outcome	Survey variant		
	DHS style	Intentional	Unintentional
**Number (percent) who agreed that intimate partner violence was justified**			
Wife goes out without permission	103 (23%)	181 (41%)	92 (21%)
Wife neglects children	96 (21%)	271 (62%)	55 (13%)
Wife argues in public	150 (33%)	294 (67%)	84 (19%)
Wife refuses sexual intercourse	76 (17%)	166 (38%)	63 (14%)
Wife does not prepare food on time	147 (32%)	296 (67%)	46 (10%)
**Number of items endorsed, mean (standard deviation)**	1.26 (1.58)	2.74 (1.81)	0.77 (1.19)

**Table 3:** Perceived norms about the acceptability of intimate partner violence, unadjusted estimates by survey condition (*n =* 1,334).

Outcome	Survey variant		
	DHS style	Intentional	Unintentional
**Number (percent) who responded that >50% of people in their village would agree that intimate partner violence was justified**			
Wife goes out without permission	155 (34%)	206 (47%)	122 (28%)
Wife neglects children	118 (26%)	241 (55%)	75 (17%)
Wife argues in public	183 (40%)	262 (60%)	132 (30%)
Wife refuses sexual intercourse	143 (31%)	181 (41%)	124 (28%)
Wife does not prepare food on time	134 (29%)	254 (58%)	68 (15%)
**Number of items endorsed, mean (standard deviation)**	1.61 (1.68)	2.59 (1.92)	1.19 (1.49)

In the multivariable partial proportional odds regression models, contextual information about the intentionality of the wife’s violations of gendered standards of behavior had a statistically significant effect on both participants’ personal beliefs and perceived norms. With the participants assigned to the DHS-style survey variant as the referent group, participants assigned to the intentional survey variant condoned intimate partner violence in a greater number of circumstances. As can be appreciated in Table 4, the magnitude of the adjusted odds ratios (AORs) increased across the thresholds in an inverted U-shaped fashion, with the largest effect observed for endorsing intimate partner violence in 3–5 vignettes (AOR = 5.74; 95% CI, 4.41–7.48; *p <* 0.001). In contrast, participants assigned to the unintentional survey variant were less likely to endorse intimate partner violence, and this inhibitory effect was greatest for the most extreme threshold, endorsing intimate partner violence in all five vignettes (AOR = 0.29; 95% CI, 0.11–0.56; *p =* 0.001).

**Table 4:** Personal beliefs about the acceptability of intimate partner violence, adjusted estimates by survey condition, using partial proportional odds regression (*n =* 1,334).

Survey variant	Endorsed intimate partner violence in 1–5 vignettes (versus no vignettes)		Endorsed intimate partner violence in 2–5 vignettes (versus 0–1 vignette)		Endorsed intimate partner violence in 3–5 vignettes (versus 0–2 vignettes)		Endorsed intimate partner violence in 4–5 vignettes (versus 0–3 vignettes)		Endorsed intimate partner violence in all five vignettes (versus 0–4 vignettes)	
	AOR (95% CI)	*p*-Value	AOR (95% CI)	*p*-Value	AOR (95% CI)	*p*-Value	AOR (95% CI)	*p*-Value	AOR (95% CI)	*p*-Value
DHS style	Ref		Ref		Ref		Ref		Ref	
Intentional	3.87 (3.05–4.91)	<0.001	4.56 (3.84–5.41)	<0.001	5.74 (4.41–7.48)	<0.001	5.18 (3.60–7.47)	<0.001	3.91 (2.37–6.44)	<0.001
Unintentional	0.70 (0.47–1.04)	0.07	0.41 (0.28–0.61)	<0.001	0.45 (0.28–0.73)	0.001	0.36 (0.20–0.68)	0.001	0.29 (0.11–0.56)	0.001

All regression models included adjustment for sex and village to account for the stratified randomization scheme.

AOR, adjusted odds ratio.

The analysis of perceived norms displayed similar patterns, but the effects were slightly smaller in magnitude (Table 5). The effect of being assigned to the intentional survey variant increased monotonically across the thresholds, such that the effect was greatest for the most extreme threshold, perceiving intimate partner violence to be normative in all five vignettes (AOR = 3.51; 95% CI, 2.23–5.53; *p <* 0.001). However, no such pattern was observed for being assigned to the unintentional survey variant, and the AORs were roughly uniform across the thresholds.

**Table 5:** Perceived norms about the acceptability of intimate partner violence, adjusted estimates by survey condition, using partial proportional odds regression (*n =* 1,334).

Survey variant	Perceived intimate partner violence to be normative in 1–5 vignettes (versus no vignettes)		Perceived intimate partner violence to be normative in 2–5 vignettes (versus 0–1 vignette)		Perceived intimate partner violence to be normative in 3–5 vignettes (versus 0–2 vignettes)		Perceived intimate partner violence to be normative in 4–5 vignettes (versus 0–3 vignettes)		Perceived intimate partner violence to be normative in all five vignettes (versus 0–4 vignettes)	
	AOR (95% CI)	*p*-Value	AOR (95% CI)	*p*-Value	AOR (95% CI)	*p*-Value	AOR (95% CI)	*p*-Value	AOR (95% CI)	*p*-Value
DHS style	Ref		Ref		Ref		Ref		Ref	
Intentional	2.05 (1.59–2.65)	<0.001	2.21 (1.66–2.95)	<0.001	2.85 (2.21–3.67)	<0.001	3.06 (2.49–3.76)	<0.001	3.51 (2.23–5.53)	<0.001
Unintentional	0.65 (0.52–0.81)	<0.001	0.63 (0.52–0.76)	<0.001	0.56 (0.44–0.72)	<0.001	0.49 (0.35–0.68)	<0.001	0.53 (0.23–1.22)	0.14

All regression models included adjustment for sex and village to account for the stratified randomization scheme.

AOR, adjusted odds ratio.

Next we assessed interactions by sex to test the hypothesis that women’s responses would be more sensitive to contextual information. Consistent with this hypothesis, we found that the magnitudes of the estimated effects differed by sex of the participant (Tables A and B in S1 Appendix). Both men and women assigned to the intentional survey variant were more likely to endorse intimate partner violence, but only for men did the effects increase in magnitude monotonically across the thresholds. In contrast, women assigned to the unintentional survey variant were less likely to endorse intimate partner violence, and this effect was strongest for the most extreme threshold of endorsing intimate partner violence in all five vignettes (AOR = 0.22; 95% CI, 0.10–0.47; *p <* 0.001). Men assigned to the unintentional survey variant were also less likely to endorse intimate partner violence, but the effects were statistically significant for only two of the five thresholds. Similar patterns were observed in the sex-stratified analysis of perceived norms: women assigned to the intentional survey variant were more likely than men to perceive intimate partner violence as normative, while women assigned to the unintentional survey variant were less likely than men to perceive intimate partner violence as normative.

Our findings were robust to alternative specifications. When we separately examined each of the five questions assessing personal beliefs and each of the five questions assessing perceived norms, the pattern of estimates was consistent with our primary findings (Tables C and D in S1 Appendix). The intentional survey variant had a much more consistent effect on increasing the probability of endorsing intimate partner violence (compared to the effect of the unintentional survey variant on decreasing the probability of endorsing intimate partner violence). Our findings were also robust to adjustment for interviewer–participant sex concordance, as the interaction between interviewer sex and participant sex was not statistically significant in the analysis of either personal beliefs (interaction AOR = 1.04; 95% CI, 0.74–1.47; *p =* 0.83) or perceived norms (interaction AOR = 1.14; 95% CI, 0.63–2.05; *p =* 0.67). Finally, when we analyzed the data using a different set of dependent variables that did not assume equal weights, we obtained qualitatively similar findings (Table E in S1 Appendix).

## Discussion

In this population-based, randomized survey experiment conducted in rural Uganda, we found that, in the measurement of personal beliefs and perceived norms about the acceptability of intimate partner violence, study participants were sensitive to contextual information about the circumstances surrounding the violence. Moreover, contextual details had larger effects on personal beliefs than on perceived norms. These effects were large in magnitude and robust to alternative specifications. Given the randomized study design, these estimates have a causal interpretation, in the sense that hearing certain contextual details in the vignettes caused people to differentially endorse personal beliefs and perceived norms about intimate partner violence. Taken together, our findings have important implications for research as well as policymaking and programmatic work in the field.

The observation that contextual details about the intentionality of the wife’s violations of gendered standards of behavior shifted participants’ personal beliefs and perceived norms about the acceptability of intimate partner violence provides novel experimental evidence from sub-Saharan Africa to corroborate qualitative research suggesting the need for better measurement in the field. In the first exploration of this phenomenon, a mixed-methods study from Bangladesh that included cognitive interviews and focus group discussions, Schuler et al. [29] showed that the extent to which study participants condoned the perpetration of violence against women was contingent on contextual details. Our study provides confirmatory experimental evidence from rural Uganda that contextual details matter for eliciting personal beliefs and perceived norms about the acceptability of intimate partner violence. Compared to the DHS-style survey variant, the survey variant depicting the wife as intentionally violating gendered standards of behavior had a “large” effect [66] in terms of increasing the number of items for which intimate partner violence was endorsed as acceptable. In contrast, the survey variant depicting the wife as unintentionally violating standards of behavior had a “small” effect in terms of decreasing the number of items endorsed. Stated more succinctly, participants’ responses depended on the extent to which they believed the women in the hypothetical vignettes to be “blameless” or “blameworthy.”

The study that is most closely related to ours is a study based on a random-digit dial sample from the US state of California [30]. Taylor and Sorenson [30] also employed a vignette design, in which they presented study participants with vignettes depicting intimate partner violence and then asked them to make attributions of fault to the victim, the assailant, or equally to both parties. Although their study was limited by a relatively low response rate (52%), experimental manipulation of up to 16 contextual variables (e.g., demographic characteristics of the victim, whether the victim and/or assailant had consumed alcohol prior to the incident) permitted them to assess the extent to which these factors were predictive of attributions of fault. A key difference is that our population-based study experimentally manipulated attributions of fault in order to elicit personal beliefs and perceived norms about whether or not intimate partner violence was justifiable in such incidents—thereby having direct relevance for design decisions about the DHS.

Interpretation of our findings is subject to two important limitations. First, as with any survey-based research, our assessments of personal beliefs and perceived norms could have been measured with error. However, random measurement error in the dependent variable would have biased our estimates towards the null. Furthermore, the very premise of this study is that personal beliefs and perceived norms about violence against women are in fact measured with error, and our study uses a randomized design to precisely identify an important source of this error. In this vein, our study contributes to a larger body of literature on survey experiments, including experiments involving the item count technique [67], list experiments [68], randomized question ordering [69], and factorial survey designs [70]. These methods have been used—primarily in resource-rich settings—to study a variety of sensitive topics, including violence against women [71,72]. Second, the study was conducted in a single geographic region of rural Uganda, potentially limiting the extrapolation of our findings to other settings. Gender-unequal norms are known to vary widely throughout sub-Saharan Africa [16,17], so it is possible that the provision of contextual details may influence survey responses differently in Uganda compared to other countries. However, our findings are consistent with qualitative research from rural Bangladesh [29], and our estimates of the extent to which there is agreement about the acceptability of intimate partner violence under particular circumstances are generally consistent with those reported in the 2011 Uganda Demographic and Health Survey [73]. Further, our survey procedures were based on a whole-population survey [74]. All of these mitigating factors suggest that our findings may generalize beyond rural Uganda.

With these limitations in mind, our findings raise an important question: how can personal beliefs and perceived norms about the acceptability of intimate partner violence be measured accurately? Relatedly, should the DHS questions on intimate partner violence—which have for nearly two decades served as an important source of worldwide data on beliefs about the acceptability intimate partner violence [26]—be revised? Our study design did not contain a survey condition in which personal beliefs and perceived norms were elicited using a criterion standard, because there is in fact no criterion standard. In the absence of a criterion standard, because the three survey conditions could be compared only to each other, it is arguably unclear which version of the questions is the most “accurate.” That being said, the DHS-style survey variant was in between the two other survey variants in terms of the mean number of items for which intimate partner violence was endorsed as acceptable: specifically, the mean number of items endorsed by study participants who were administered the DHS-style survey variant was closer to the mean number of items endorsed by study participants who were administered the unintentional survey variant. This pattern of outcomes suggests that DHS-style questions—in which women’s motivations are left unspecified—may yield biased estimates if most people, when presented with such vignettes, make assumptions about the extent to which the woman is “blameless” or “blameworthy.” There is a well-known surfeit of data—albeit from North American and European settings—suggesting that victim-blaming attitudes are relatively prevalent [75–77], particularly among perpetrators of violence [78,79]. Thus, an interesting follow-up research question would be to assess the extent to which survey respondents arrive at causal attributions when presented with vignettes in which intentionality is not mentioned, as in the DHS. Such research would be important in helping to inform whether and how the DHS questions should be changed.

## Supporting information

S1 CONSORT Checklist (DOC)

S1 Appendix (DOCX)

## Abbreviations

AOR: adjusted odds ratio
DHS: Demographic and Health Surveys
