# Supplementary material for: Measuring personal beliefs and perceived norms about intimate partner violence: Population-based survey experiment in rural Uganda
Source: PLoS Med. 2017 May 23;14(5):e1002303. doi: 10.1371/journal.pmed.1002303 (PMC5441576; doi:10.1371/journal.pmed.1002303)
Supplement: S1 Appendix — (DOCX) [file pmed.1002303.s002.docx]

**Text A.** Survey questions to elicit personal beliefs and perceived norms about acceptability of intimate partner violence

*Version 1: Provides little contextual detail, similar to those included in the Demographic and Health Surveys*

Now I would like to ask you some questions about the way husbands may behave with regards to their wives. It is normal for a couple to have quarrels and disagreements. During these quarrels, some husbands may reprimand their wives with words or beat their wives physically. Remember there are no wrong answers.

| In your personal opinion, do you agree or disagree with the following statement? |
| --- |
| 1. A husband is justified in hitting or beating his wife if she goes out without telling him or asking his permission. |
| 1. A husband is justified in hitting or beating his wife if she neglects to keep the children well fed. |
| 1. A husband is justified in hitting or beating his wife if she argues with him in public. |
| 1. A husband is justified in hitting or beating his wife if she refuses to have sexual intercourse with him. |
| 1. A husband is justified in hitting or beating his wife if she does not prepare her husband’s meal on time. |

Now I will ask you whether MOST other people aged 18 years or older who stay in your village, not including yourself, would agree with the statements, according to your perception. I understand that you may not know the exact beliefs of most other people who stay in your village, but please do your best to answer. Remember there are no wrong answers.

| How many other people aged 18 years or older who stay in your village, not including yourself, do you think would agree with the following statement? |
| --- |
| 1. A husband is justified in hitting or beating his wife if she goes out without telling him or asking his permission. |
| 1. A husband is justified in hitting or beating his wife if she neglects to keep the children well fed. |
| 1. A husband is justified in hitting or beating his wife if she argues with him in public. |
| 1. A husband is justified in hitting or beating his wife if she refuses to have sexual intercourse with him. |
| 1. A husband is justified in hitting or beating his wife if she does not prepare her husband’s meal on time. |

*Version 2: Depicts the wife as intentionally violating gendered standards of behavior*

Now I would like to ask you some questions about the way husbands may behave with regards to their wives. It is normal for a couple to have quarrels and disagreements. During these quarrels, some husbands may reprimand their wives with words or beat their wives physically. Remember there are no wrong answers.

| In your personal opinion, do you agree or disagree with the following statement? |
| --- |
| 1. Imagine a situation in which a wife goes out to visit some friends or relatives, just for fun, without asking her husband’s permission or telling him where she is going. In this situation, the husband would be justified in hitting or beating his wife. |
| 1. Imagine a situation in which a wife regularly permits her children to walk around the village looking like they do not have enough to eat. The husband has asked her several times before to keep them well fed, but she does not pay attention to what he asks. In this situation, the husband would be justified in hitting or beating his wife. |
| 1. Imagine a situation in which a wife often contradicts her husband in the presence of others, and argues with him frequently in public. In this situation, the husband would be justified in hitting or beating his wife. |
| 1. Imagine a situation in which a wife does not wish to have sexual intercourse with her husband. In this situation she is not menstruating, she is not pregnant, and she does not suspect her husband of having relations with other women. In this situation, the husband would be justified in hitting or beating his wife. |
| 1. Imagine a situation in which a wife spends much of her day gossiping with her friends. Because of this, even though the husband has generated income for the family and brought her what she needs to prepare a meal, she regularly serves her husband’s meal late. In this situation, the husband would be justified in hitting or beating his wife. |

Now I will ask you whether MOST other people aged 18 years or older who stay in your village, not including yourself, would agree with the statements, according to your perception. I understand that you may not know the exact beliefs of most other people who stay in your village, but please do your best to answer. Remember there are no wrong answers.

| How many other people aged 18 years or older who stay in your village, not including yourself, do you think would agree with the following statement? |
| --- |
| 1. Imagine a situation in which a wife goes out to visit some friends or relatives, just for fun, without asking her husband’s permission or telling him where she is going. In this situation, the husband would be justified in hitting or beating his wife. |
| 1. Imagine a situation in which a wife regularly permits her children to walk around the village looking like they do not have enough to eat. The husband has asked her several times before to keep them well fed, but she does not pay attention to what he asks. In this situation, the husband would be justified in hitting or beating his wife. |
| 1. Imagine a situation in which a wife often contradicts her husband in the presence of others, and argues with him frequently in public. In this situation, the husband would be justified in hitting or beating his wife. |
| 1. Imagine a situation in which a wife does not wish to have sexual intercourse with her husband. In this situation she is not menstruating, she is not pregnant, and she does not suspect her husband of having relations with other women. In this situation, the husband would be justified in hitting or beating his wife. |
| 1. Imagine a situation in which a wife spends much of her day gossiping with her friends. Because of this, even though the husband has generated income for the family and brought her what she needs to prepare a meal, she regularly serves her husband’s meal late. In this situation, the husband would be justified in hitting or beating his wife. |

*Version 3: Depicts the wife as unintentionally violating gendered standards of behavior*

Now I would like to ask you some questions about the way husbands may behave with regards to their wives. It is normal for a couple to have quarrels and disagreements. During these quarrels, some husbands may reprimand their wives with words or beat their wives physically. Remember there are no wrong answers.

| In your personal opinion, do you agree or disagree with the following statement? |
| --- |
| 1. Imagine a situation in which a wife is home alone, and the husband is away at work. Someone comes to tell her that her mother is very ill, so she goes to her parents’ home without asking her husband’s permission and stays the night. In this situation, the husband would be justified in hitting or beating his wife upon her return. |
| 1. Imagine a situation in which a wife regularly keeps her children well fed. Today the well ran dry so she has to spend much time obtaining water from another source. Because she is so busy, at dinner there is less food and the children go to bed hungry. In this situation, the husband would be justified in hitting or beating his wife. |
| 1. Imagine a situation in which a man stays home out of laziness for several days, refusing to go out and look for work. One day they are together in public, and his wife tells him they are running out of food and asks him to look for work so they can have enough money to buy more food. This leads to an argument in public. In this situation, the husband would be justified in hitting or beating his wife. |
| 1. Imagine a situation in which a husband has been staying out late and often comes home smelling of alcohol. The wife suspects her husband of having relations with other women, and because she fears getting a sexually transmitted disease, she refuses to have sex with him. In this situation, the husband would be justified in hitting or beating his wife. |
| 1. Imagine a situation in which a wife is usually very busy doing the housework and tending to the children. Normally she manages to complete her work, but today things were busier than usual and she served her husband’s meal late. In this situation, the husband would be justified in hitting or beating his wife. |

Now I will ask you whether MOST other people aged 18 years or older who stay in your village, not including yourself, would agree with the statements, according to your perception. I understand that you may not know the exact beliefs of most other people who stay in your village, but please do your best to answer. Remember there are no wrong answers.

| How many other people aged 18 years or older who stay in your village, not including yourself, do you think would agree with the following statement? |
| --- |
| 1. Imagine a situation in which a wife is home alone, and the husband is away at work. Someone comes to tell her that her mother is very ill, so she goes to her parents’ home without asking her husband’s permission and stays the night. In this situation, the husband would be justified in hitting or beating his wife upon her return. |
| 1. Imagine a situation in which a wife regularly keeps her children well fed. Today the well ran dry so she has to spend much time obtaining water from another source. Because she is so busy, at dinner there is less food and the children go to bed hungry. In this situation, the husband would be justified in hitting or beating his wife. |
| 1. Imagine a situation in which a man stays home out of laziness for several days, refusing to go out and look for work. One day they are together in public, and his wife tells him they are running out of food and asks him to look for work so they can have enough money to buy more food. This leads to an argument in public. In this situation, the husband would be justified in hitting or beating his wife. |
| 1. Imagine a situation in which a husband has been staying out late and often comes home smelling of alcohol. The wife suspects her husband of having relations with other women, and because she fears getting a sexually transmitted disease, she refuses to have sex with him. In this situation, the husband would be justified in hitting or beating his wife. |
| 1. Imagine a situation in which a wife is usually very busy doing the housework and tending to the children. Normally she manages to complete her work, but today things were busier than usual and she served her husband’s meal late. In this situation, the husband would be justified in hitting or beating his wife. |

**Table A.** Personal beliefs about the acceptability of intimate partner violence, adjusted estimates by survey condition and stratified by sex, using partial proportional odds regression (N=1,334)

|  | Endorsed intimate partner violence in 1-5 vignettes (vs. no vignettes) | | | Endorsed intimate partner violence in 2-5 vignettes (vs. 0-1 vignette) | | | Endorsed intimate partner violence in 3-5 vignettes (vs. 0-2 vignettes) | | | Endorsed intimate partner violence in 4-5 vignettes (vs. 0-3 vignettes) | | | Endorsed intimate partner violence in all 5 vignettes (vs. 0-4 vignettes) | | |
| --- | --- | --- | --- | --- | --- | --- | --- | --- | --- | --- | --- | --- | --- | --- | --- |
|  | AOR | (95% CI) | P-value | AOR | (95% CI) | P-value | AOR | (95% CI) | P-value | AOR | (95% CI) | P-value | AOR | (95% CI) | P-value |
| *Women* |  |  |  |  |  |  |  |  |  |  |  |  |  |  |  |
| Survey variant |  |  |  |  |  |  |  |  |  |  |  |  |  |  |  |
| Ver. 1, DHS-style | Ref | Ref |  | Ref | Ref |  | Ref | Ref |  | Ref | Ref |  | Ref | Ref |  |
| Ver. 2, Intentional | 4.72 | (3.75-5.95) | <0.001 | 5.40 | (3.62-8.05) | <0.001 | 6.32 | (3.71-10.7) | <0.001 | 4.90 | (2.82-8.51) | <0.001 | 3.46 | (2.27-5.30) | <0.001 |
| Ver. 3, Unintentional | 0.58 | (0.36-0.92) | 0.02 | 0.32 | (0.22-0.47) | <0.001 | 0.35 | (0.19-0.62) | <0.001 | 0.34 | (0.18-0.64) | 0.001 | 0.22 | (0.10-0.47) | <0.001 |
|  |  |  |  |  |  |  |  |  |  |  |  |  |  |  |  |
| *Men* |  |  |  |  |  |  |  |  |  |  |  |  |  |  |  |
| Survey variant |  |  |  |  |  |  |  |  |  |  |  |  |  |  |  |
| Ver. 1, DHS-style | Ref | Ref |  | Ref | Ref |  | Ref | Ref |  | Ref | Ref |  | Ref | Ref |  |
| Ver. 2, Intentional | 3.39 | (2.02-5.68) | <0.001 | 4.10 | (2.60-6.45) | <0.001 | 5.58 | (3.52-8.85) | <0.001 | 6.12 | (4.07-9.20) | <0.001 | 5.75 | (1.76-18.8) | 0.004 |
| Ver. 3, Unintentional | 0.91 | (0.64-1.30) | 0.62 | 0.60 | (0.36-0.99) | 0.046 | 0.69 | (0.37-1.27) | 0.23 | 0.41 | (0.18-0.95) | 0.037 | 0.34 | (0.03-3.97) | 0.39 |

All regression models included adjustment for sex and village to account for the stratified randomization scheme

**Table B.** Perceived norms about the acceptability of intimate partner violence, adjusted estimates by survey condition and stratified by sex, using partial proportional odds regression (N=1,334)

|  | Perceived intimate partner violence to be normative in 1-5 vignettes (vs. no vignettes) | | | Perceived intimate partner violence to be normative in 2-5 vignettes (vs. 0-1 vignette) | | | Perceived intimate partner violence to be normative in 3-5 vignettes (vs. 0-2 vignettes) | | | Perceived intimate partner violence to be normative in 4-5 vignettes (vs. 0-3 vignettes) | | | Perceived intimate partner violence to be normative in all 5 vignettes (vs. 0-4 vignettes) | | |
| --- | --- | --- | --- | --- | --- | --- | --- | --- | --- | --- | --- | --- | --- | --- | --- |
|  | AOR | (95% CI) | P-value | AOR | (95% CI) | P-value | AOR | (95% CI) | P-value | AOR | (95% CI) | P-value | AOR | (95% CI) | P-value |
| *Women* |  |  |  |  |  |  |  |  |  |  |  |  |  |  |  |
| Survey variant |  |  |  |  |  |  |  |  |  |  |  |  |  |  |  |
| Ver. 1, DHS-style | Ref | Ref |  | Ref | Ref |  | Ref | Ref |  | Ref | Ref |  | Ref | Ref |  |
| Ver. 2, Intentional | 3.16 | (2.49-4.02) | <0.001 | 3.06 | (1.86-5.02) | <0.001 | 3.72 | (2.57-5.38) | <0.001 | 3.69 | (2.67-5.11) | <0.001 | 4.10 | (2.53-6.64) | <0.001 |
| Ver. 3, Unintentional | 0.75 | (0.56-1.01) | 0.057 | 0.71 | (0.52-0.97) | 0.03 | 0.54 | (0.37-0.80) | 0.002 | 0.45 | (0.30-0.68) | 0.45 | 0.50 | (0.21-1.16) | 0.11 |
|  |  |  |  |  |  |  |  |  |  |  |  |  |  |  |  |
| *Men* |  |  |  |  |  |  |  |  |  |  |  |  |  |  |  |
| Survey variant |  |  |  |  |  |  |  |  |  |  |  |  |  |  |  |
| Ver. 1, DHS-style | Ref | Ref |  | Ref | Ref |  | Ref | Ref |  | Ref | Ref |  | Ref | Ref |  |
| Ver. 2, Intentional | 1.31 | (0.71-2.41) | 0.39 | 1.53 | (0.97-2.39) | 0.07 | 2.05 | (1.49-2.80) | <0.001 | 2.40 | (1.46-3.95) | 0.001 | 2.85 | (1.38-5.90) | 0.005 |
| Ver. 3, Unintentional | 0.54 | (0.39-0.74) | <0.001 | 0.54 | (0.35-0.85) | 0.007 | 0.62 | (0.38-1.04) | 0.07 | 0.48 | (0.23-1.46) | 0.25 | 0.62 | (0.15-2.62) | 0.52 |

All regression models included adjustment for sex and village to account for the stratified randomization scheme

**Table C.** Personal beliefs about the acceptability of intimate partner violence, adjusted estimates by treatment arm using Poisson regression (N=1,334)

| Abuse justified if wife… | …goes out without permission | | | …neglects children | | | …argues in public | | | …refuses sexual intercourse | | | …does not prepare food on time | | |
| --- | --- | --- | --- | --- | --- | --- | --- | --- | --- | --- | --- | --- | --- | --- | --- |
|  | ARR | (95% CI) | P-value | ARR | (95% CI) | P-value | ARR | (95% CI) | P-value | ARR | (95% CI) | P-value | ARR | (95% CI) | P-value |
| Survey variant |  |  |  |  |  |  |  |  |  |  |  |  |  |  |  |
| Ver. 1, DHS-style | Ref |  |  | Ref |  |  | Ref |  |  | Ref |  |  | Ref |  |  |
| Ver. 2, Intentional | 1.81 | (1.48-2.23) | <0.001 | 2.90 | (2.67-3.16) | <0.001 | 2.01 | (1.79-2.25) | <0.001 | 2.25 | (1.71-2.97) | <0.001 | 2.06 | (1.83-2.31) | <0.001 |
| Ver. 3, Unintentional | 0.93 | (0.67-1.28) | 0.67 | 0.60 | (0.44-0.81) | 0.001 | 0.58 | (0.43-0.78) | <0.001 | 0.86 | (0.65-1.14) | 0.30 | 0.32 | (0.23-0.46) | <0.001 |

All regression models included adjustment for sex and village to account for the stratified randomization scheme

**Table D.** Perceived norms about the acceptability of intimate partner violence, adjusted estimates by treatment arm using Poisson regression (N=1,334)

| More than half of people in village would agree that abuse is justified if wife… | …goes out without permission | | | …neglects children | | | …argues in public | | | …refuses sexual intercourse | | | …does not prepare food on time | | |
| --- | --- | --- | --- | --- | --- | --- | --- | --- | --- | --- | --- | --- | --- | --- | --- |
|  | ARR | (95% CI) | P-value | ARR | (95% CI) | P-value | ARR | (95% CI) | P-value | ARR | (95% CI) | P-value | ARR | (95% CI) | P-value |
| Survey variant |  |  |  |  |  |  |  |  |  |  |  |  |  |  |  |
| Ver. 1, DHS-style | Ref |  |  | Ref |  |  | Ref |  |  | Ref |  |  | Ref |  |  |
| Ver. 2, Intentional | 1.37 | (1.21-1.55) | <0.001 | 2.09 | (1.73-2.53) | <0.001 | 1.47 | (1.35-1.60) | <0.001 | 1.30 | (1.12-1.51) | 0.001 | 1.94 | (1.84-2.05) | <0.001 |
| Ver. 3, Unintentional | 0.82 | (0.71-0.94) | 0.005 | 0.66 | (0.47-0.93) | 0.02 | 0.75 | (0.66-0.85) | <0.001 | 0.90 | (0.84-0.96) | 0.001 | 0.53 | (0.42-0.65) | <0.001 |

All regression models included adjustment for sex and village to account for the stratified randomization scheme

**Table E.** Perceived norms about the acceptability of intimate partner violence, using dependent variables derived from principal components analysis *, adjusted estimates by treatment arm using linear regression (N=1,334)

|  | IPV beliefs index | | | IPV perceived norms index | | |
| --- | --- | --- | --- | --- | --- | --- |
|  | b | (95% CI) | P-value | b | (95% CI) | P-value |
| Survey variant |  |  |  |  |  |  |
| Ver. 1, DHS-style | Ref |  |  | Ref |  |  |
| Ver. 2, Intentional | 1.44 | (1.27-1.62) | <0.001 | 0.93 | (0.74-1.13) | <0.001 |
| Ver. 3, Unintentional | -0.47 | (-0.73 to -0.21) | 0.004 | -0.41 | (-0.57 to -0.25) | 0.001 |

* Dependent variables based on the total count of items implicitly assume equal weights for the component variables. Here we conduct a sensitivity analysis using a different set of dependent variables that do not assume equal weights. We use principal components analysis to determine the weights for an index of the 5 IPV outcome variables (Filmer D, Pritchett L (1999) The effect of household wealth on educational attainment: evidence from 35 countries. Popul Dev Rev 25:85-120). This method is a technique that can be applied to a set of variables to extract the orthogonal linear combinations of the variables that represent the common information (in this case, a latent construct measuring belief in gender-unequal norms). Intuitively, the first principal component is simply the linear combination of all the variables that captures the largest amount of information that is common to the variables. The weights are not grounded theoretically but rather empirically. We extract the first principal components and label them our dependent variables of interest (“IPV beliefs index” and “IPV perceived norms index”). The IPV beliefs index has a mean of 0 and a standard deviation of 1.70, with a range from -1.54 to 3.29. The IPV perceived norms index has a mean of 0 and a standard deviation of 1.69, with a range from -1.68 to 2.99. The absolute values are meaningless but can be used to rank order study participants in terms of their beliefs and perceptions about gender-unequal norms. All regression models in this table included adjustment for sex and village to account for the stratified randomization scheme.
